# Biomarker-targeted functionalized magnetic nanoparticles: synthesis and aptamer conjugation optimization toward Alzheimer's disease biosensing

**DOI:** 10.1039/d6na00021e

**Published:** 2026-04-06

**Authors:** Antonios Makridis, Konstantina Kazeli, Georgios Katsipis, Eleni E. Tzekaki, Anastasia A. Pantazaki, Cristian Bosch, Ricardo Simon-Carbajo, Makis Angelakeris

**Affiliations:** a Magnetic Nanostructure Characterization: Technology & Applications, Centre for Interdisciplinary Research and Innovation, Aristotle University Thessaloniki 57001 Greece anmakrid@physics.auth.gr; b Department of Condensed Matter and Materials Physics, School of Physics, Aristotle University of Thessaloniki Thessaloniki 54124 Greece; c Laboratory of Neurodegenerative Diseases, Centre for Interdisciplinary Research and Innovation, Aristotle University Thessaloniki 57001 Greece; d Laboratory of Biochemistry, Department of Chemistry, Aristotle University of Thessaloniki Thessaloniki 54124 Greece; e Ireland's Centre for Artificial Intelligence (CeADAR), University College Dublin Belfield D04 V2N9 Dublin Ireland

## Abstract

Magnetic-plasmonic hybrid nanoparticles are gaining prominence in biomedical diagnostics due to their dual functionality, combining magnetic manipulation and signal enhancement. A major challenge remains the reproducible synthesis of core–shell nanostructures with controlled size, composition, and stability. In this work, we present a robust two-step aqueous co-precipitation method to produce gold/magnetite nanoparticles, for early Alzheimer's disease diagnosis, based on biomarker's aptamer-based biosensing. Magnetite nanoparticles were first synthesized with high saturation magnetization, followed by controlled gold shell growth *via* citrate-assisted reduction. Systematic tuning of gold precursor ratios and washing steps enabled precise control over the shell structure and surface properties. The nanoparticles were extensively characterized using X-ray diffraction, dynamic light scattering, zeta potential analysis, vibrating sample magnetometry, ultraviolet-visible spectroscopy, and inductively coupled plasma measurements, outlining optimal characteristics: distinct core–shell morphology, ferrimagnetic behavior, strong localized surface plasmon resonance, and high colloidal stability. Beyond synthesis and characterization of nanoparticles, this study also introduces an innovative aptamer conjugation protocol tailored for maximizing their binding efficiency, thereby enhancing early recognition of certain neurodegenerative biomarkers: Aβ-40, Aβ-42, TBA and GFAP. The tailored features of the gold/magnetite nanoparticles allow fine control over particle size and aptamer loading, making this work a valuable tool for designing structurally, magnetically, and physicochemically optimized carriers for neurodegenerative disease diagnostics. Collectively, these results establish a scalable and functional platform suitable for next-generation biosensing applications in the early detection of Alzheimer's disease.

## Abstract 2

Optimized synthesis and aptamer functionalization of hybrid Au@Fe nanoparticles provide a stable magnetic-plasmonic platform with high loading capacity and reproducibility for Alzheimer's disease biomarker detection.

## Graphical abstract

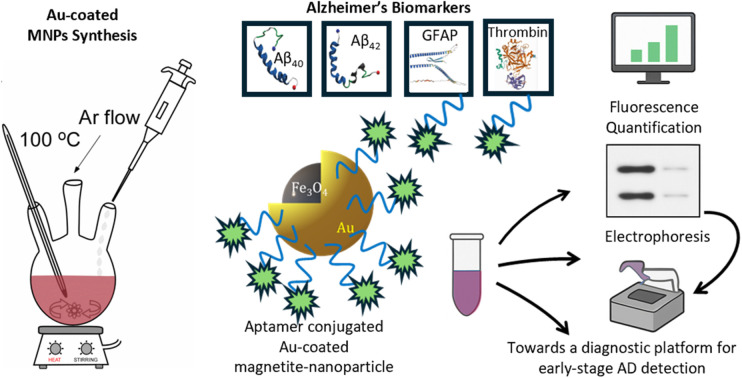

## 1 Introduction

The development of multifunctional nanomaterials has opened new avenues in biomedical research, enabling integrated platforms for diagnosis, targeting, and therapy.^1–3^ Among noble metal nanostructures, gold nanoparticles are widely recognized for their excellent biocompatibility, chemical stability, and highly tunable surface chemistry.^2^ Their strong affinity with thiol, disulfide, amine, and carboxyl groups allows for straightforward conjugation with biomolecules such as peptides, antibodies, and DNA aptamers.^4–6^ These attributes render gold-based nanostructures ideal candidates for targeted biosensing and molecular recognition.^7^

In parallel, iron oxide nanoparticles—particularly magnetite (Fe_3_O_4_)—have been extensively investigated owing to their strong magnetic responsiveness, high saturation magnetization, and biocompatibility. Such properties enable their use in applications including magnetic separation,^8^ MRI contrast enhancement, magnetically guided drug delivery,^9^ and magnetic hyperthermia-based therapies.^10^ Among the various synthetic strategies, aqueous co-precipitation remains one of the most effective and scalable routes for producing Fe_3_O_4_ nanoparticles, although their surface reactivity and colloidal stability often require additional functionalization for biological compatibility.^9,11^

Integrating these two systems into a single core–shell architecture—comprising a magnetic Fe_3_O_4_ core coated with a gold shell (Au@Fe)—combines the magnetic manipulation capability of iron oxide with the surface versatility and plasmonic properties of gold.^11,12^ This hybrid structure supports precise magnetic control while providing an accessible and chemically stable gold surface for biomolecular conjugation. Nonetheless, challenges persist in achieving homogeneous gold coatings, ensuring reproducibility, and retaining long-term colloidal stability in aqueous media.

To address these limitations, we developed a two-step aqueous synthesis strategy for Au@Fe nanoparticles, employing controlled citrate-assisted reduction alongside systematic optimization of the precursor concentration, magnetic separation timing, and washing procedure. This approach yielded reproducible nanostructures with strong plasmonic response, high saturation magnetization, and excellent colloidal stability, establishing a robust platform for biofunctionalization.

Alzheimer's disease (AD) remains a chronic, progressive neurodegenerative disorder and the leading cause of dementia worldwide, imposing a profound socio-economic burden.^13^ Although existing cerebrospinal fluid biomarkers—such as reduced Aβ_42_, altered Aβ_42_/Aβ_40_ ratio, and elevated phosphorylated tau—support diagnosis, delays and diagnostic uncertainty remain common. Thrombin, a main driver of the coagulation cascade, has been identified as important in pathological events in AD and other neurodegenerative diseases.^14^ Emerging biomarkers, including glial fibrillary acidic protein (GFAP) and neurofilament light chain (NfL) have gained attention for reflecting astroglial activation, axonal injury, and neurovascular dysfunction, respectively.^15,16^

Ultrasensitive immunochemical platforms, such as Simoa (Single Molecule Array—a digital immunoassay technology developed by Quanterix and a leading platform for NfL quantification), along with automated assays, enable the precise measurement of these biomarkers, driving the development of portable and cost-effective diagnostic tools.^17^ However, significant challenges persist, including the establishment of universal cut-off values, the accurate differentiation of Alzheimer's Disease (AD) from other neurodegenerative disorders, and the integration of multiplexed biomarker panels—potentially enhanced by machine learning—to optimize early detection and longitudinal monitoring.^18^

Beyond conventional recognition elements, oligonucleotide aptamers have emerged as highly promising molecular binders due to their exceptional sensitivity, chemical stability, and ease of modification.^19–21^ Next-generation biosensing technologies that combine nanomaterials with molecular recognition elements—such as antibodies and aptamers—have gained significant traction.^22–26^ These biofunctional nanoplatforms exploit electrochemical, plasmonic, SERS-based, fluorescence-based, and colorimetric transduction mechanisms to detect key AD biomarkers with increasing sensitivity and selectivity.^27–30^ Amyloid-β peptides (Aβ_40_ and Aβ_42_), particularly their oligomeric forms, have been a primary focus of research, and several DNA and RNA aptamers have been developed for both their detection and inhibition of aggregation.^23,31–33^

First described in 1990 as RNA ligands with high affinity for small molecules and T4 DNA polymerase,^34,35^ aptamers have since been explored extensively for diagnostic and therapeutic applications, including AD.^22,36–38^ Their binding ability arises from distinct secondary and tertiary structures capable of forming hydrogen bonds, hydrophobic contacts, electrostatic interactions, van der Waals interactions, steric complementarity, and π–π stacking.^39^ Their compact size facilitates blood–brain barrier penetration,^40–42^ while their chemical synthesis ensures high purity, low batch variability, and cost effectiveness.^43^ Aptamers exhibit dissociation constants (*K*_D_) ranging from picomolar to micromolar,^39,44–48^ and although off-target binding may occur, their stability and specificity have earned them the label “chemical antibodies”.^49^

Compared to antibodies, aptamers offer simplified assay integration: they can be used in homogeneous format or immobilized on polymeric surfaces, carbon nanostructures, or nanoparticles through electrostatic, hydrophobic, covalent, or thiol–gold interactions, circumventing the multistep coupling protocols typically required for antibodies.^38,50^ Moreover, their solid-phase chemical synthesis aligns with the 3 Rs principle, offering a fully animal-free route to high-quality reagents.^51,52^

Recent advances highlight biofunctional magnetic nanoparticles (MNPs) as potent agents for both diagnostic and therapeutic strategies.^19,33^ In this context, we sought to establish a reliable aptamer-nanoparticle conjugation workflow for tailor-made Au@Fe magnetic nanoparticles. Using reference sequences, we evaluated the conjugation behavior of aptamers relevant to Alzheimer's disease biomarkers, including the Aβ7-92-1H1 aptamer, which has been reported to inhibit Aβ42 aggregation, the RNV95 aptamer, known for its high affinity toward Aβ oligomers, and the thrombin-binding aptamer Thrombin-Binding Aptamer 1 (TBA1).^30,31^ Following optimization of buffer composition, ionic strength, aptamer dilution strategy, and washing conditions, we extended the protocol to functionalize the nanoparticles with a SELEX-derived aptamer targeting GFAP (FIB1C-T3).

In this study, we report the aqueous synthesis, structural characterization, and magnetic evaluation of Au@Fe nanoparticles engineered for aptamer conjugation and biosensing applications. Systematic optimization of gold-shell growth, precursor stoichiometry, and surface charge enabled the identification of an optimal formulation exhibiting high colloidal stability, strong magnetic responsiveness, and well-defined plasmonic activity, which served as the foundation for the subsequent development of the biosensing platform. Leveraging these properties, we functionalized the nanoparticles with a thiolated DNA aptamer targeting major AD biomarkers, including Aβ_42_, Aβ_40_, thrombin, and GFAP. The resulting biofunctional nanoplatform integrates magnetic separation with molecular recognition, laying the foundation for next-generation aptamer-based biosensing systems capable of selective, multiplexed, and rapid detection of neurodegenerative disease biomarkers.

## 2 Experimental

### 2.1 Materials

Iron(iii) chloride hexahydrate (FeCl_3_·6H_2_O, 99%) and iron(ii) chloride tetrahydrate (FeCl_2_·4H_2_O, 99%) were purchased from Panreac (Darmstadt, Germany). Ammonia solution (25%) and tri-sodium citrate dihydrate (Na_3_C_6_H_5_O_7_·2H_2_O, 99%) were obtained from VWR Chemicals (Leuven, Belgium). Tetrachloroauric(III) acid trihydrate (HAuCl_4_·3H_2_O, 99.5%) was supplied by Chem-Lab (Zedelgem, Belgium). All chemicals were used without further purification. Distilled water was used in all procedures.

### 2.2 Synthetic procedures

The synthesis of Au@Fe was performed *via* a two-step protocol adapted from Mühlberger *et al.*,^53^ Elbialy *et al.*,^54^ and Stein *et al.*,^55^ with critical modifications introduced in both the magnetic core synthesis and gold-coating process.

### 2.3 Step 1: synthesis of Fe_3_O_4_ nanoparticles

Fe_3_O_4_ nanoparticles were synthesized *via* alkaline co-precipitation of ferric and ferrous chlorides, adapting the mass ratio protocol described by Elbialy *et al.*^54^ Specifically, FeCl_3_·6H_2_O (1.622 g) and FeCl_2_·4H_2_O (0.9941 g) (representing a mass ratio of 1.63 : 1) were dissolved in 40 mL of deionized water under an argon atmosphere to prevent oxidation. While stirring vigorously with a magnetic stirrer at high speed to prevent localized aggregation, 5.6 mL of 25% NH_3_ solution was added dropwise, resulting in the immediate formation of a black ferrofluid characteristic of magnetite (Fe_3_O_4_).

**Fig. 1:**
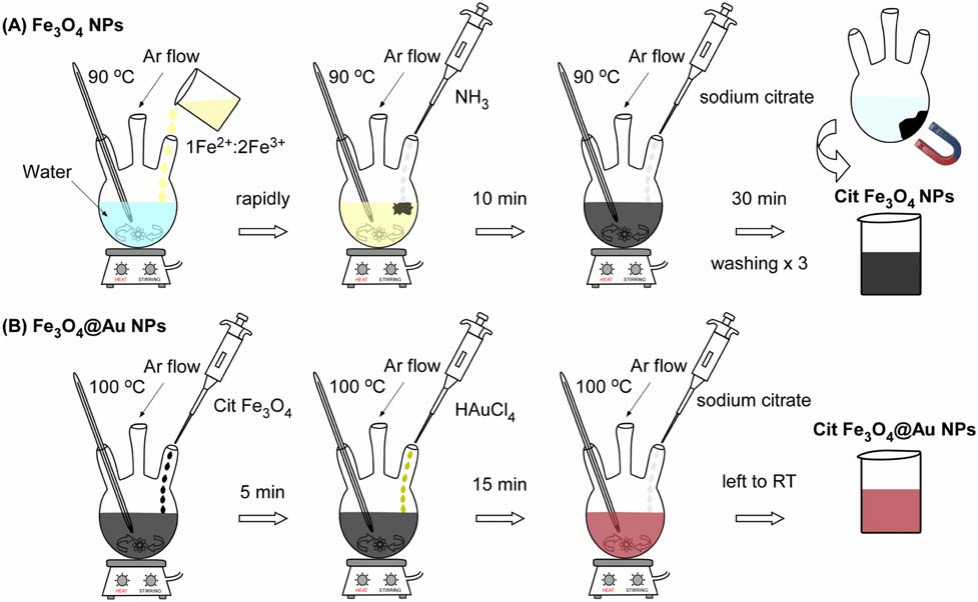
Schematic representation of the two-step synthesis of citric acid-functionalized Au@Fe nanoparticles. (a) In the first step, magnetite (Fe_3_O_4_) nanoparticles are synthesized and coated with citric acid to enhance stability and dispersibility. (b) In the second step, a gold (Au) shell is formed on the Fe_3_O_4_ cores in the presence of additional citric acid, which acts as a stabilizing and capping agent, yielding fully functionalized Au@Fe nanoparticles.

After 10 minutes of stirring, 15 mL of a 0.3 mg mL^−1^ sodium citrate solution was added to the dispersion. The mixture was then heated to 90 °C under vigorous stirring and maintained at this temperature for 30 minutes to facilitate citrate functionalization, as illustrated in Fig. 1. Afterward, the dispersion was allowed to cool to RT. Five different formulations of citrate-stabilized magnetic nanoparticles were developed (Fe-1, Fe-2, … Fe-5). These samples served as the precursor cores for the subsequent gold-coating process in Step 2, which yielded the final hybrid Au@Fe-1 to Au@Fe-5 nanostructures.

### 2.4 Post-synthesis treatments

Au@Fe-1: after citrate stabilization, the nanoparticles were magnetically separated and redispersed in distilled water to an iron concentration of 13.2 mg mL^−1^, following the protocol reported by Stein *et al.*^55^ An aliquot of 35 mL of this stock dispersion was subsequently diluted with deionized water to a final reaction volume of 80 mL for the gold coating step.

Au@Fe-2: the crude ferrofluid (110 mL) was used directly. From this, an 80 mL aliquot was taken for gold deposition without prior magnetic separation.

Au@Fe-3, Au@Fe-4, Au@Fe-5: the nanoparticles were magnetically separated, redispersed in 50 mL of distilled water, and then diluted 1 : 20 (v/v). From these diluted dispersions, 80 mL aliquots were used for the gold coating step.

### 2.5 Step 2: gold coating of Fe_3_O_4_ nanoparticles

For the gold coating, 80 mL of the diluted Fe_3_O_4_ colloidal dispersion was transferred to a three-neck flask and heated to 100 °C under continuous and vigorous magnetic stirring to ensure homogeneity. A freshly prepared 20 mL aqueous solution of HAuCl_4_ was rapidly added under high-speed magnetic stirring. The HAuCl_4_ concentrations used for each formulation were 0.2 mg mL^−1^ for Au@Fe-1, Au@Fe-2, and Au@Fe-3; 0.4 mg mL^−1^ for Au@Fe-4; and 0.6 mg mL^−1^ for Au@Fe-5. These variations in the gold precursor concentration, combined with the post-synthesis treatments, aimed to investigate their influence on nanoparticle morphology and coating efficiency.

The reaction mixtures were maintained at 100 °C for 15 minutes, during which a color change from brown to wine-red was observed, indicating successful gold deposition. Subsequently, 1 mL of a freshly prepared aqueous solution containing 26 mg of tri-sodium citrate dihydrate was added to the mixture and stirred for 30 seconds to provide final capping and stabilization. The dispersion was then allowed to cool to RT and stored at 4 °C for further characterization. The two-step synthesis and the corresponding color transitions are schematically illustrated in Fig. 1. In the first step, magnetite (Fe_3_O_4_) nanoparticles are synthesized and coated with citric acid to enhance stability and dispersibility. In the second step, a gold (Au) shell is formed on the Fe_3_O_4_ cores in the presence of additional citric acid, which acts as a stabilizing and capping agent, yielding fully functionalized Au@Fe nanoparticles. Representative colors of the dispersions at the end of each synthetic step are shown in Fig. 1, where the citrate-stabilized Fe_3_O_4_ nanoparticles exhibit a characteristic black color. In contrast, the Au@Fe nanoparticles display a wine-red hue due to the localized surface plasmon resonance of the gold shell. It is important to note that while the Fe_3_O_4_ cores were thoroughly purified *via* magnetic separation and washing in the first step to remove unreacted precursors, the final Au@Fe nanoparticles were maintained as a stable colloidal dispersion without further washing. This approach preserves the integrity of the citrate capping layer, which is essential for the colloidal stability and LSPR properties of the gold shell. The successful formation of the shell is macroscopically confirmed by the transition from the characteristic black color of the magnetite cores to a deep wine-red hue.

### 2.6 Characterization

The structure of the nanoparticles was determined by X-ray diffraction (XRD) with a Bruker D8 Advance X-ray powder diffractometer using Cu K_α_ radiation at 40 kV and 25 mA using the Kα line of Cu as a radiation source in the 2*Θ* range from 15° to 70° with a scanning step width of 0.02° and 0.4 s scanning time per step. The magnetic properties were measured by using a vibrating sample (1.2H/CF/HT Oxford Instruments VSM).

Particle size analysis was carried out by dynamic light scattering (DLS). An SZ-100V2 HORIBA particle size analyzer was used for particle size and colloidal stability measurement, equipped with a diode-pumped frequency-doubled laser light source (*λ* = 532 nm), with a dynamic range of 0.3 nm–10 µm. Since DLS is based on Brownian motion, not gravitational settling, the upper limit is determined using sample density, while the lower limit is impacted by concentration, the intensity of the scattered light beam, and the presence of large area particles. The SZ-100 measured the suspension's zeta potential to determine the charge on the surface of the particles. The nanoparticles were dispersed in ultra-pure water at a low concentration (0.1 mg mL^−1^), and the pH was adjusted to 7.0 to ensure a consistent chemical environment for all formulations. This was followed by ultrasonication for 10 min to achieve homogeneity. The samples were injected into 5 mL disposable cuvettes, and the calculated zeta potential was obtained by measuring the electrophoretic mobility of the particles. The samples' measured zeta potential at pH 7.0 is utilized as a metric of dispersion stability.

The absorption spectra of aqueous Au@Fe nanoparticle dispersions with a concentration of 25 µg Fe mL^−1^ were measured using an ultraviolet-visible (UV-Vis) spectrophotometer (Libra S22, Biochrom, Cambridge, United Kingdom). The measurement range was set between 250 and 700 nm with a step size of 2 nm.

### 2.7 Molecular docking simulations

Molecular docking simulations were conducted to gain structural insight into the molecular recognition mechanisms and to support the experimentally determined binding affinities of the RNV95, Aβ7-92-1H1, and TBA1 aptamers. AI-assisted analysis, specifically utilizing the SPPIDER tool,^56^ aids biomarker selection and interaction site prediction by integrating multimodal datasets and identifying subtle structural or sequence patterns. This approach, which leverages transformer-based language models (such as ESM-2) to analyze protein and aptamer sequences, was used to prioritize candidates according to their predictive relevance and to define active residues for docking.

Subsequently, docking was performed using HADDOCK 2.4 (High Ambiguity Driven protein-protein DOCKing).^57,58^ The workflow involved three main stages: preparation of structures, including optimization and definition of active and passive residues; execution of docking through rigid-body docking, semi-flexible refinement, and final refinement in explicit solvent; and, finally, scoring and clustering of the resulting complexes. Predicted structures were grouped based on root-mean-square deviation (RMSD), producing clusters that summarize the most representative binding conformations.

For each cluster, HADDOCK reports key parameters such as the average HADDOCK score, which reflects interaction strength and favors more negative values; the *Z*-score, indicating how strongly a cluster deviates from the mean of all solutions; the cluster size, which reflects sampling convergence; and the buried surface area (BSA), quantifying the molecular interface formed after docking. These metrics collectively enable comparative evaluation of binding modes and interaction quality across the aptamer-target systems. It should be noted that while molecular docking provided structural insights for RNV95, Aβ7-92-1H1, and TBA1, simulations were not conducted for the fib1c-t3 aptamer. This specific lead was identified and validated *via* SELEX by a partner within the 2D-BioPAD consortium. Due to the proprietary nature of its nucleotide sequence, fib1c-t3 was incorporated into the study based on its established experimental binding affinity toward GFAP, which precluded its inclusion in the current computational modeling workflow.

### 2.8 Aptamers and conjugation on Au@Fe nanoparticles

The employed aptamers for this study were for Aβ_40_: RNV95 (5′-TGGGGGGCGGACGATAGGGGCCCCCCGGTAGGATGGACG-3′), Aβ7-92-1H1 for Aβ_42_: (5′-CCGGTGGGGGACCAGTACAAAAGTGGGTAGGGCGGGTTGGAAAA-3′), for thrombin: TBA1 (5′-GGTTGGTGTGGTTGG-3′), and for GFAP: FIB1C-T3. RNV95, Aβ7-92-1H1 and TBA1 aptamer sequences were as proposed by Chakravarthy *et al.*,^32^ Zhang *et al.*,^59^ and Bock *et al.*,^60^ respectively. The aptamer FIB1C-T3 was selected by an aptamer specialist company, Novaptech.^61^ All aptamers were synthesized by Microsynth AG (Balgach, Switzerland), carrying 5′-C6-S-S (thiol) modification. For quantitation studies, a 3′-6-FAM (fluorescein) modification was also added. Initially, aptamers were activated by mixing with 10 µM dithiothreitol (DTT) for 1 h, at RT. Then, DTT was removed from the mixture with three subsequent washes with Tris-EDTA (TE) buffer, pH 8.0, through Amicon® Ultra 0.5 mL centrifugal filters with a cut-off of 3 kDa (UFC500324). Finally, aptamers were diluted with TE to a concentration of 100 µM.

The protocol used for successfully conjugating thiol-modified aptamers was the freezing-directed method according to Liu and Liu,^62^ with some modifications that were verified in the current study, as described in the sections below. The final employed protocol was as follows: 0.32 mg mL^−1^ of Au@Fe nanoparticles were mixed with SH-aptamer at a final volume of 500 µL, in sterile centrifuge tubes. To determine the molar stoichiometry, the molecular weight of the Au@Fe nanoparticles was estimated at 2.27 10^8^ g mol^−1^ based on their size and composition (detailed calculation provided in Section S.1 of the SI). This corresponds to a nanoparticle molar concentration of 1.4 nM, ensuring a significant molar excess of aptamers (1–2 µM) to promote efficient surface functionalization and maximal coverage. The optimized added concentrations for each aptamer were 1 µM for TBA1 and FIB1C-T3 and 2 µM for RNV95 and Aβ7-92-1H1. Aptamers were mixed for 15 min with MNPs at room temperature (RT) and then 10 mM citrate buffer, pH 3, was added. Tubes were mixed and rested for 15 min, followed by overnight incubation at −20 °C. The following day, mixtures were thawed at RT in the darkness and centrifuged at 10 000 rpm, for 15 min at 4 °C, followed by 4 washes with TE buffer to discard the unbound aptamer. All samples were either resuspended in dd H_2_O to obtain photographs or further analyzed immediately after washing stages, to ensure the reproducibility and integrity of our results. In all cases, the samples were protected from light and kept at a maximum room temperature, to minimize detachment or degradation. Sterile buffers were used in all cases.

### 2.9 Determination of the bound aptamer

To quantify the attached aptamer in each case, FAM-modified aptamers were employed. Aptamer-Au@Fe-4 nanoconjugates were treated with 0.1 M DTT for 5 min at 50 °C, followed by 1 h of incubation at RT, in the dark. After centrifugation at 14 000 rpm for 15 min, at 4 °C, the quantity of the bound aptamer was determined with the fluorescence measurement of the supernatant (excitation at 490 nm; emission at 510–570 nm) using a Promega GloMax Multi detection system. The concentration of the aptamers was determined with standard curves designed with FAM-modified aptamer dilutions.

### 2.10 Optimization of aptamer conjugation

Optimization of the conjugation protocol was performed using the Aβ7-92-1H1 aptamer for Aβ42. During conjugation, we evaluated the following Au@Fe-4 final concentrations: 0.08, 0.16, 0.32 and 0.64 mg mL^−1^. The following buffers were evaluated in terms of increasing the conjugation efficiency of the studied aptamers on Au@Fe-4: (i) TE buffer 10 mM, pH 8, (ii) citrate sodium buffer 10 mM, pH 3, and (iii) HEPES sodium buffer 10 mM, pH 7.6. Sterile dd H_2_O was also used as a reference. In addition, we investigated whether salt addition (30, 50, 100, 200, or 500 mM of NaCl) improves or impairs aptamer conjugation. For this evaluation, 0.32 mg mL^−1^ of Au@Fe-4 were mixed with 1 µM Aβ7-92-1H1, in a conjugation buffer of citrate sodium (pH 3). The studied buffers were evaluated as (a) conjugation buffers during the overnight incubation of the aptamer-Au@Fe-4 mixes and/or (b) washing buffers to remove the unbound or weakly bound aptamers from Au@Fe-4 after conjugation. In the first test, 0.16 mg mL^−1^ of Au@Fe-4 were mixed with one of the studied buffers and the aptamer, and after overnight incubation, washing steps were performed with TE buffer (pH 8). In the second test, 0.32 mg mL^−1^ of Au@Fe-4 were mixed with the aptamer and the optimized citrate buffer (pH 3). After overnight incubations, washings were performed with one of the studied buffers. All experiments were performed under the same conditions to obtain comparable and replicable results.

### 2.11 Agarose gel electrophoresis

To determine the electrophoretic motility of the Au@Fe nanoparticles after conjugation with the Aβ7-92-1H1 aptamer, the centrifuged aptamer-Au@Fe nanoconjugates were resuspended in a buffer containing 5 mM HEPES (pH 7.6) and 50% glycerol. Samples were loaded and then electrophoresed in a 0.8% (w/v) agarose gel for 30 min at 80 V.

### 2.12 Statistical analysis

All experiments were run at least in independent triplicate and were analyzed using one-way ANOVA followed by Tukey's post hoc test. Differences were considered statistically significant at *p* < 0.05. Graph Pad Prism 8 was used for all analyses.

## 3 Results and discussion

### 3.1 Structural, optical, and magnetic characterization of Au@Fe nanoparticles

**Fig. 2:**
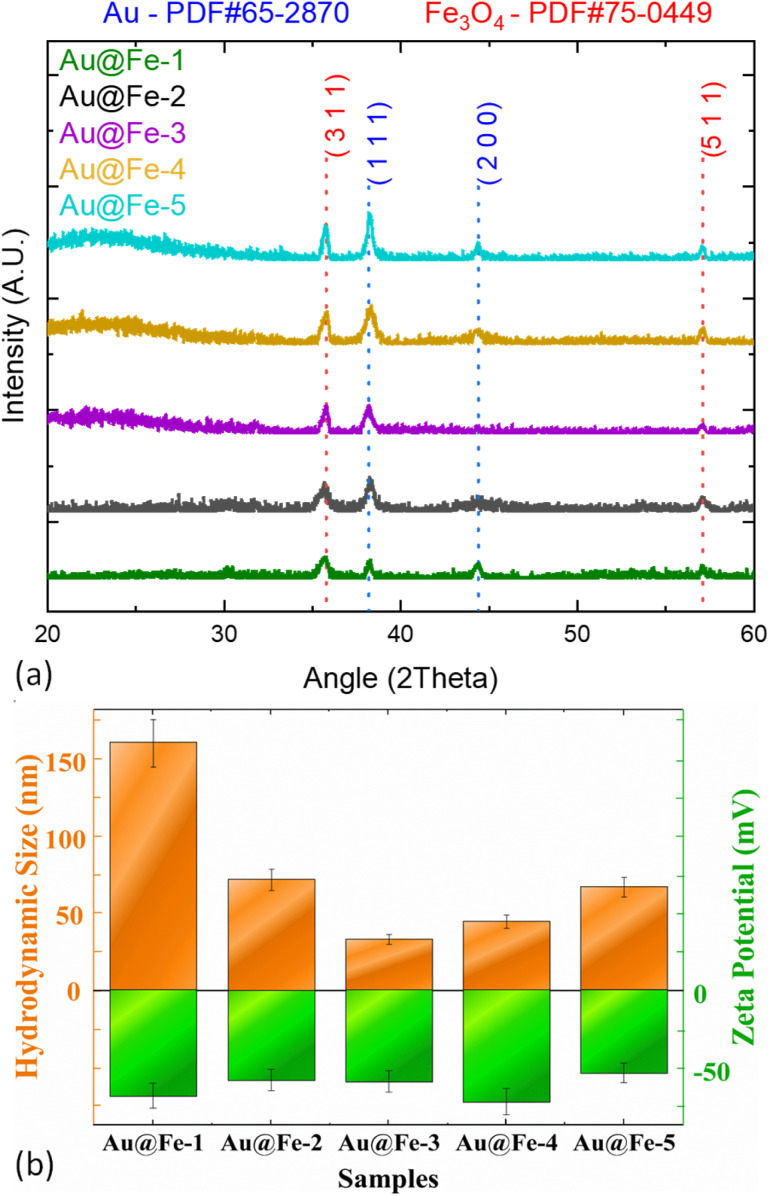
Characterization of Au@Fe nanoparticles across five samples (Au@Fe-1 to Au@Fe-5). (a) XRD patterns showing the characteristic peaks of magnetite (Fe_3_O_4_) and gold (Au), indexed to PDF 75-0449 and PDF 65-2870, respectively. The increasing intensity of gold-related peaks from Au@Fe-3 to Au@Fe-5 indicates enhanced deposition of the gold shell with a higher HAuCl_4_ concentration. (b) DLS measurements illustrate changes in the hydrodynamic diameter (orange bars) and surface charge (green bars) across the samples. Notably, sample Au@Fe-3 exhibits the smallest hydrodynamic diameter (∼33 nm), while Au@Fe-4 displays the most negative ζ-potential (−73 mV), indicating a very high surface charge magnitude and excellent electrostatic stability. In addition, Au@Fe-4 shows well-defined gold diffraction peaks in the XRD pattern, confirming successful Au shell formation.

XRD analysis (Fig. 2a) confirmed the successful synthesis of Au@Fe nanoparticles across all five samples (Au@Fe-1 to Au@Fe-5). All diffraction patterns clearly show the characteristic peaks of both magnetite (Fe_3_O_4_) and gold (Au), indexed to PDF 75-0449 and PDF 65-2870, respectively. The XRD patterns of all Au@Fe formulations (Fig. 2a) were normalized to the (311) diffraction peak of the Fe_3_O_4_ phase to facilitate a comparative analysis of the shell-to-core signal ratio. As observed, while the magnetite reflections remain consistent, a progressive increase in the intensity of the Au (111) and (200) peaks is evident from Au@Fe-1 to Au@Fe-5, correlating with the higher gold precursor loading used during synthesis. As expected, the intensity of the gold-related peaks increases progressively from sample Au@Fe-3 to Au@Fe-5, consistent with the increasing Au concentration (as per the HAuCl_4_ concentration used during synthesis). This trend reflects the denser deposition of the gold shell onto the magnetite core with a higher excess gold precursor.

DLS-derived hydrodynamic size and zeta potential measurements (Fig. 2b) provide complementary information on the colloidal properties and surface functionalization of the samples. The hydrodynamic diameter and surface charge (orange and green bars in Fig. 2b, respectively) reveal the influence of the synthetic parameters on the colloidal behavior of the nanoplatforms. While sample Au@Fe-3 exhibits the smallest hydrodynamic size (∼33 nm), it displays relatively weak gold diffraction peaks in the XRD pattern. In contrast, Au@Fe-4 was identified as the optimal formulation, as it combines a small size (∼45 nm) with the highest negative zeta potential (∼−73 mV) among all samples.

This high surface charge indicates a dense citrate coating, ensuring exceptional electrostatic stability and providing ample functional groups for aptamer attachment. The difference in zeta potential between Au@Fe-3 (−60 mV) and Au@Fe-4 (−73 mV) is significant, reflecting a more robust stabilization that correlates with the well-defined gold reflections and the characteristic wine-red hue observed for this sample. The progressive color evolution across the entire Au@Fe series (Au@Fe-1 to Au@Fe-5) is visually documented in Fig. S1 (SI). While the initial formulations exhibit a darker brownish hue, the transition to a vibrant wine-red color in Au@Fe-3 and Au@Fe-4 provides macroscopic evidence of the successful gold shell formation and its associated LSPR, consistent with the single-sample photograph shown in Fig. 4, inset.

Such a color variation, attributable to the LSPR of the gold shell, serves as a strong visual indicator of effective gold coating in good agreement with the structural and colloidal analysis. Although Au@Fe-3 presents the smallest particle size, Au@Fe-4 offers the best overall balance between small hydrodynamic size, highly negative ζ-potential, and well-defined gold shell formation, making it the most suitable formulation for subsequent functionalization studies.

The physicochemical properties, including the hydrodynamic diameter, ζ-potential, and XRD peak intensities for the entire series (Au@Fe-1 to Au@Fe-5), are summarized in Table S1 (SI). Based on the comprehensive physicochemical screening (Table S1), Au@Fe-4 was identified as the optimal candidate due to its superior colloidal stability and shell integrity. Consequently, all subsequent functionalization studies, including ICP-OES elemental analysis and aptamer binding quantification, were focused exclusively on this formulation to standardize the biosensing protocol. While the hydrodynamic sizes ranged from 33 nm to 185 nm, Au@Fe-4 was identified as the optimal candidate. Although Au@Fe-3 exhibited the smallest size (∼33 nm), it showed lower colloidal stability and less-defined gold crystallinity compared to Au@Fe-4, which combined a small size (45 nm) with the highest negative surface charge (−73 mV) and superior shell formation.

**Fig. 3:**
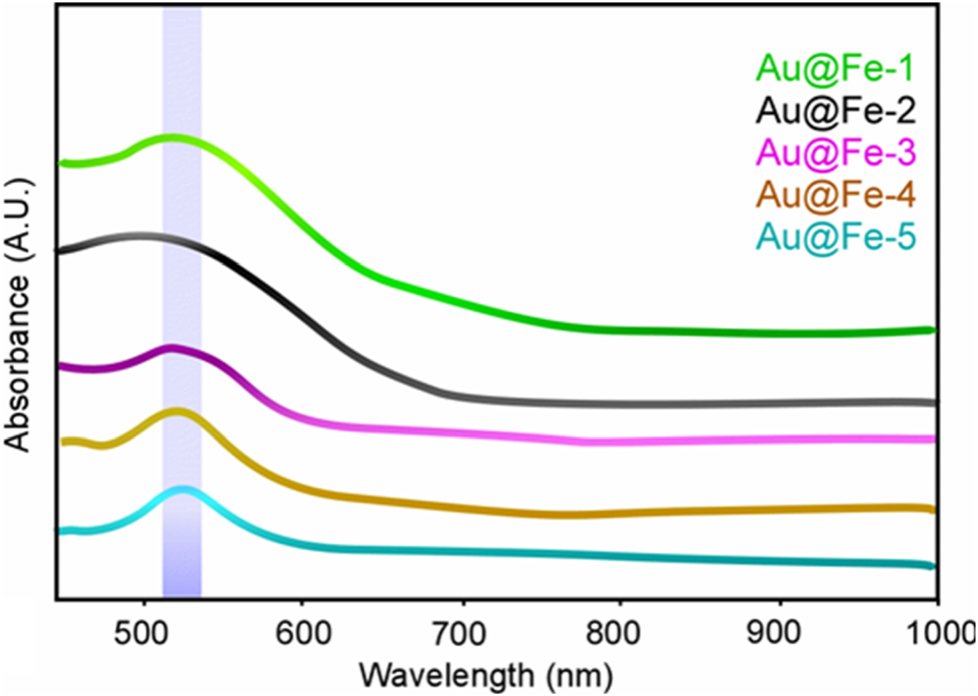
UV-Vis absorption spectra of Au@Fe nanoparticles (Au@Fe-1–Au@Fe-5). The light blue shaded region highlights the characteristic LSPR band of gold, observed between 520–540 nm. A progressive sharpening and intensification of the LSPR peak is seen from Au@Fe-3 to Au@Fe-5, indicating increasing gold shell coverage. In contrast, samples Au@Fe-1 and Au@Fe-2 exhibit broader and weaker SPR signals due to the dominant presence of magnetite and a lower effective gold-to-magnetite ratio, which diminishes the plasmonic response. These results confirm the successful formation of gold shells and demonstrate the tunability of optical properties through controlled synthesis.

DLS revealed that Au@Fe-4 exhibits a hydrodynamic diameter of 45 nm—ideal for surface bioconjugation—and the most negative ζ-potential of −73 mV, indicating exceptional colloidal stability and resistance to aggregation. The UV-Vis spectra (Fig. 3) demonstrated a well-resolved LSPR peak at ∼530 nm; the absence of a significant red-shift in this peak confirms that the nanoparticles remained monodisperse and that the gold shell thickness was uniform across the batches. The optical properties and the presence of Localized Surface Plasmon Resonance (LSPR) were evaluated to confirm the successful formation of the gold shell. The formation of the gold shell was spectroscopically confirmed *via* UV-Vis analysis (Fig. 3), where all formulations displayed a prominent LSPR absorption band at 530 nm. The symmetry and intensity of this peak, particularly for the optimized Au@Fe-4 sample, correlate with the successful deposition of a continuous gold layer and the observed wine-red coloration. This resonance feature, which is absent in bare magnetite cores, provides macroscopic and spectroscopic evidence of the hybrid core–shell architecture. This spectroscopic evidence, combined with the XRD and DLS data, justifies the selection of Au@Fe-4 as the optimal formulation for subsequent evaluation in magnetic sensing and aptamer-based biochemical assays. In addition, to provide definitive structural proof of this hybrid architecture, Transmission Electron Microscopy (TEM) was employed (Fig. S2, SI). The TEM images of Au@Fe-4 reveal a decorated hybrid morphology where the magnetite nanoparticles are successfully integrated with larger gold nanostructures, forming stable clusters. The TEM-derived dimensions (approximately 40 nm) are in excellent agreement with the hydrodynamic diameter of 45 nm measured by DLS for the Au@Fe-4 formulation. This cross-validation between TEM and DLS confirms that the nanoparticles remain monodisperse and that the gold phase is effectively coupled with the magnetic core rather than existing as a separate phase.

Magnetic characterization was performed for all five Au@Fe nanoparticle formulations to evaluate whether the variation in the gold precursor concentration and magnetic separation conditions affected their magnetic response. The magnetic hysteresis loops of all samples exhibited soft ferromagnetic behavior characterized by low coercivity and rapid saturation, which is typical for magnetite-based nanoparticles.

Only minor variations in the saturation magnetization (*M*_s_) were observed among the different formulations. These differences are attributed to the varying amounts of gold deposited on the nanoparticle surface, resulting from the different concentrations of chloroauric acid used during synthesis. Since the saturation magnetization is expressed in emu g^−1^ (per gram of total nanoparticle mass), an increased gold content leads to a slight decrease in the measured *M*_s_ due to the non-magnetic contribution of the gold phase.

Overall, the magnetic measurements confirm that all Au@Fe nanoparticle formulations preserve the intrinsic magnetic behavior of the magnetite core. For this reason, the magnetic hysteresis loops of the entire nanoparticle series are provided in the SI (Fig. S3), while Au@Fe-4—identified as the most suitable formulation based on hydrodynamic size distribution, ζ-potential, and optical response—was selected for further functionalization and sensing experiments.

**Fig. 4:**
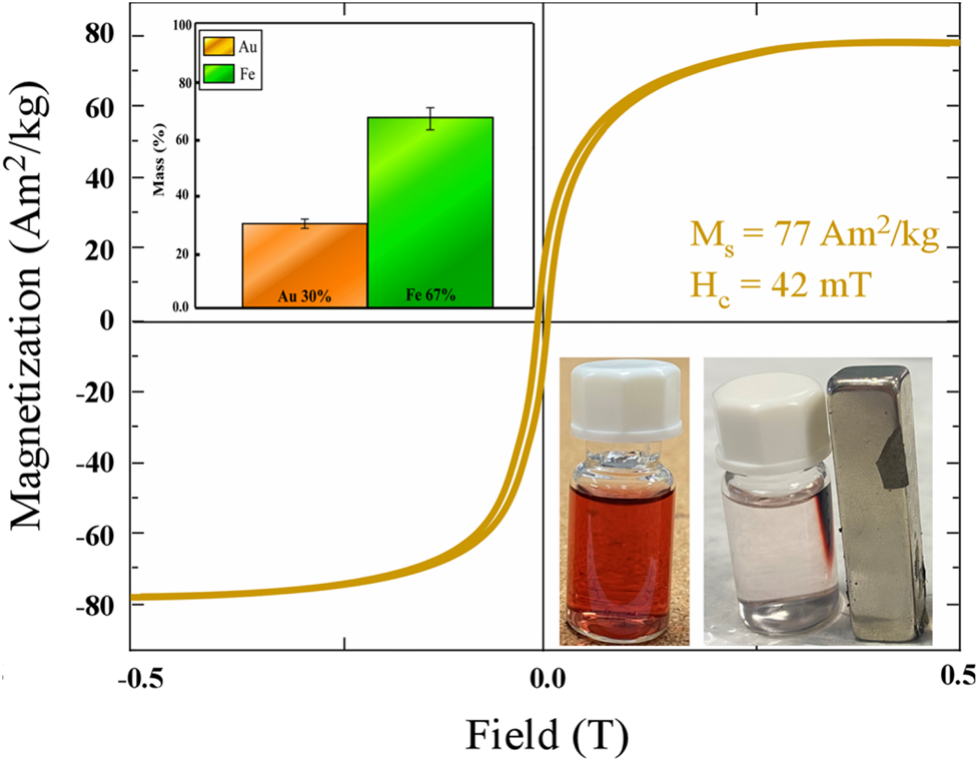
Magnetic hysteresis loop of the Au@Fe-4 sample measured at room temperature, demonstrating its ferromagnetic behavior. The inset in the second quadrant presents the Inductively Coupled Plasma (ICP)-derived elemental composition, showing the relative weight percentages of Fe and Au in the nanoparticle sample. Non-metallic components, including oxygen from the magnetite lattice and citrate surface ligands, are not quantified. The inset in the fourth quadrant shows images of the nanoparticle dispersion before (left) and after (right) exposure to a permanent magnet placed at the side of the vial for a few minutes. The magnetic nanoparticles migrate toward the magnet, forming a visible accumulation, while the remaining solution becomes transparent—illustrating the strong magnetic responsiveness and efficient separation capability of the Au@Fe nanoparticles.

The magnetic characterization of the optimized Au@Fe-4 sample further reinforces its potential as a magnetically enhancing nanoplatform. The measured saturation magnetization (77 A m^2^ kg^−1^), as shown in Fig. 4, is sufficiently high to ensure strong responsiveness to external magnetic fields, enabling efficient magnetic manipulation and separation in biosensing assays. At the same time, the observed coercivity (42 mT) demonstrates a ferromagnetic behavior that is beneficial for maintaining stable magnetic interactions without significant thermal demagnetization under physiological conditions.

Following the initial physicochemical screening, ICP-OES analysis was performed on the selected Au@Fe-4 formulation to accurately determine its elemental composition and Fe/Au mass ratio. This quantitative step was essential for standardizing the subsequent magnetic sensing and aptamer-based assays. For the remaining formulations (Au@Fe-1, 2, 3, and 5), the relative efficiency of gold coating was sufficiently monitored through the comparative XRD intensities and LSPR-induced color changes (as detailed in Table S1 and Fig. S1). The top inset (Fig. 4) shows the ICP-derived elemental composition of the Au@Fe-4 nanoparticles, expressed as the relative weight percentages of Fe and Au and calculated based on a measured nanoparticle concentration of 0.17 mg mL^−1^; both oxygen from the magnetite lattice and citrate-derived surface ligands are not detected by ICP and therefore are not included in the elemental mass balance, resulting in a minor residual fraction associated with non-metallic components. The bottom inset photograph (Fig. 4) visually confirms this property, as the nanoparticles migrate rapidly towards the external NdFeB magnet, resulting in a transparent supernatant. This facile magnetic separation is a crucial feature for biosensing workflows, where rapid isolation and washing steps are required to minimize background noise and enhance assay specificity.

In combination with the strong plasmonic response and high colloidal stability demonstrated earlier, these magnetic results highlight the synergistic nature of the Au@Fe design. The magnetic core allows for efficient handling and manipulation of nanoparticles, while the gold shell provides optical readout and robust aptamer bioconjugation interfaces. Importantly, the balance between magnetization strength and colloidal stability achieved in Au@Fe-4 suggests that the material is particularly well-suited for complex biological environments, where aggregation, non-specific binding, and weak separation often undermine assay performance.

Consequently, this optimized sample was employed in all subsequent experiments involving aptamer conjugation and biochemical analyses. In the following sections, we investigate the interaction of these with thiol-modified DNA aptamers targeting AD-related biomarkers, focusing on conjugation efficiency, nanoparticle stability, and the influence of physicochemical parameters on biosensing performance.

### 3.2 Conjugation reaction of thiol-modified aptamers onto Au@Fe nanoparticles

The conjugation of thiol-modified aptamers on Au@Fe-4 MNPs was evaluated using an already established overnight freezing protocol.^62^ Based on this protocol, we further explored the possibility of optimizing conjugation efficiency by employing different buffers for the conjugation and washing steps and by increasing the salinity during conjugation and the concentration of the Au@Fe-4 MNPs on the Aβ7-92-1H1 aptamer.

#### 3.2.1 Conjugation optimization: pH environment

**Fig. 5:**
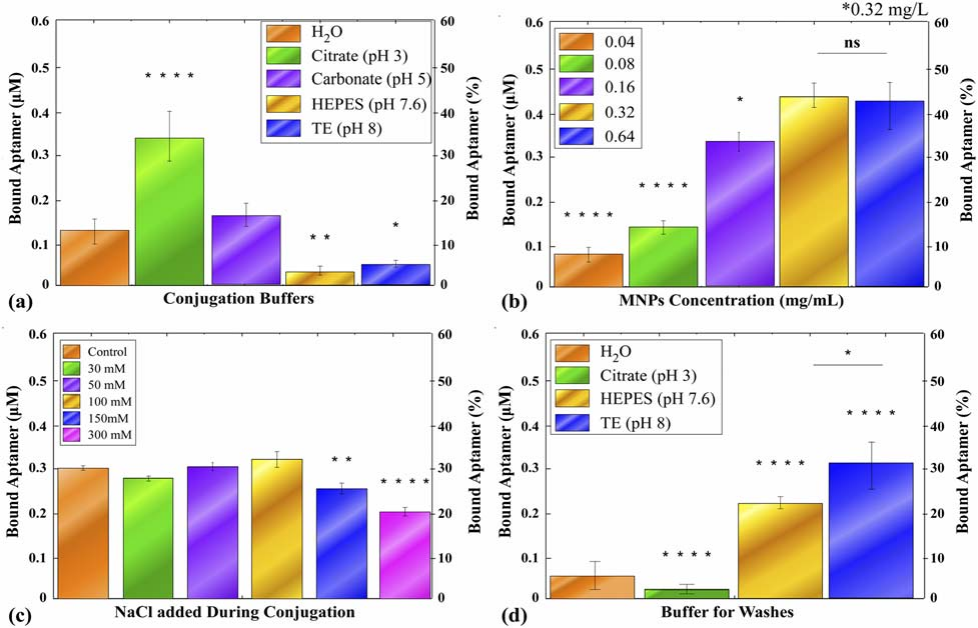
Parameters' optimization in conjugation of 1 µM DNA-aptamer Aβ7-92-1H1 to Au@Fe-4 MNPs. The bound aptamer was quantified by detaching the conjugated concentration with DTT and measuring the fluorescence of the FAM group. The quantification of the FAM-conjugated aptamer to µM was conducted using a standard curve. A secondary *y*-axis (right) is included to display the percentage of the bound aptamer, complementing the primary axis (left), which reports the absolute concentration of the bound aptamer in µM. (a) Conjugation buffer screening. Aptamer binding to Au@Fe-4 MNPs (0.16 mg mL^−1^) was assayed after conjugation under different buffer conditions (H_2_O; 10 mM citrate buffer, pH 3; 10 mM carbonate buffer, pH 5; 10 mM HEPES, pH 7.6; TE buffer, pH 8). Citrate (pH 3) markedly enhanced conjugation efficiency (****p < 0.0001 *vs.* dd H_2_O). Carbonate (pH 5) and dd H_2_O appeared to have little implication in binding efficiency. On the downside, TE (pH 8) and HEPES (pH 7.4) impeded binding affinity (**p < 0.01; *p < 0.05, respectively, when compared to incubation in no-buffer/H_2_O). (b) Screening of the nanoparticle concentration. Increasing the concentration of Au@Fe-4 MNPs from 0.04 to 0.32 mg mL^−1^ resulted progressively in higher aptamer loading. (c) Effect of ionic strength during conjugation. Aptamer binding was measured after the addition of increasing concentrations of NaCl (30–300 mM) to the conjugation mixture (citrate buffer, pH 3). Moderate salt concentrations (30–100 mM) did not impair loading, whereas 150 mM to 300 mM NaCl significantly reduced aptamer binding (**p < 0.01; ****p < 0.0001 *vs.* no-salt control). (d) Optimization of wash buffer. After the conjugation step, particles were washed in citrate (pH 3), HEPES (pH 7.6), or TE (pH 8) buffer. Low-pH citrate washes stripped off most aptamers (****p < 0.0001 *vs.* dd H_2_O wash), whereas HEPES (pH 7.6) and TE (pH 8) washes preserved or even enhanced aptamer loading (****p < 0.0001 *vs.* dd H_2_O; *p < 0.05 *vs.* TE).

To assess the effect of buffer type and pH on conjugation efficiency, Au@Fe-4 nanoparticles were incubated with DNA-aptamer Aβ7-92-1H1, and either citrate-Na_3_ (pH 3), carbonate (pH 5), HEPES-Na (pH 7.6), or Tris-EDTA (pH 8) buffer was added. After overnight incubation, conjugated aptamers on nanoparticles were detached and quantified, with dd H_2_O serving as the control. The results indicate that notable differences are observed in nanoparticle behavior under varying pH conditions (Fig. 5a). The highest affinity of conjugation was observed at pH 3 (*p* < 0.0001, compared to the control). The incubation of thiolate aptamers with Au@Fe-4 MNPs in the presence of an acidic carbonate buffer of pH 5.0 did not differentiate the conjugation efficiency in a statistically significant manner. When alkaline buffers of pH 7.6 or 8.0 were employed, the conjugation efficiency was significantly lower than that of the control (*p* < 0.01 and *p* < 0.05, respectively).

#### 3.2.2 Conjugation optimization: Au@Fe-4 MNP concentration

To investigate the effect of MNPs' concentration on the interaction between Aβ7-92-1H1 and Au@Fe-4 MNPs, we examined a concentration range of 0.04 to 0.64 mg mL^−1^ as a potential influencing parameter. Our results indicated that conjugation efficiency reaches a maximum of approximately 0.4 µM, at 0.32 mg mL^−1^ of Au@Fe-4 MNPs (Fig. 5b). This was found to be significantly higher than the conjugation efficiency when employing 0.04, 0.08, or 0.16 mg mL^−1^ of Au@Fe-4 MNPs. When the concentration of Au@Fe-4 MNPs increased further (0.64 mg mL^−1^), no concomitant significant increase in conjugation efficiency was verified. Based on the calculated molecular weight of the nanoparticles (*M*_r_ = 2.27 × 10^8^ g mol^−1^), as described in the S1 Nanoparticle Molecular Weight Calculation section of the SI, a concentration of 0.32 mg mL^−1^ corresponds to approximately 1.4 nM nanoparticles. Therefore, all aptamers were used in large molar excess relative to nanoparticles to promote efficient surface functionalization.

#### 3.2.3 Conjugation optimization: buffer salinity

To study the possible effect of ionic strength during aptamer conjugation, an addition of 30–300 mM of NaCl was followed after mixing the DNA aptamer with Au@Fe-4 MNPs and citrate buffer, pH 3. After adding citrate buffer, samples were incubated for 15 min, and conjugation efficiency was determined after overnight freezing. As depicted in Fig. 5c, for salt addition up to 100 mM, no statistically significant increase in conjugation efficiency was documented. On the other hand, the addition of high salt concentrations (150 or 300 mM) significantly decreased the conjugation efficiency (*p* < 0.01 and *p* < 0.0001, respectively). It is hypothesized that high salinity leads to unstable Au@Fe-4 MNPs' dispersity during conjugation, impeding the linking of thiolate aptamers on Au.^63–65^ Consequently, we chose not to add further NaCl during conjugation; no additional NaCl was added other than the sodium inherently present in the citrate buffer, as this ionic strength was found to be sufficient for aptamer-nanoparticle binding.

#### 3.2.4 Conjugation optimization: wash buffer

To evaluate the influence of different wash buffers on aptamer binding, washes were conducted after the overnight treatment to remove the unbound aptamer, comparing citrate buffer (pH 3.0), HEPES (pH 7.4), and TE buffer (pH 8.0) against dd H_2_O as a control (Fig. 5d). Among the tested conditions, TE buffer yielded the highest aptamer binding concentration (∼0.4 µM), which was statistically significant compared to the control (*p* < 0.0001) and the second most efficient buffer, namely HEPES (*p* < 0.05). In contrast, citrate buffer resulted in the lowest binding affinity, also showing a significant difference (*p* < 0.0001). Based on these findings, the TE buffer at pH 8 was selected as the standard wash buffer for all subsequent experiments.

### 3.3 The effect of aptamer conjugation on the electrophoretic mobility of Au@Fe-4 nanoparticles

**Fig. 6:**
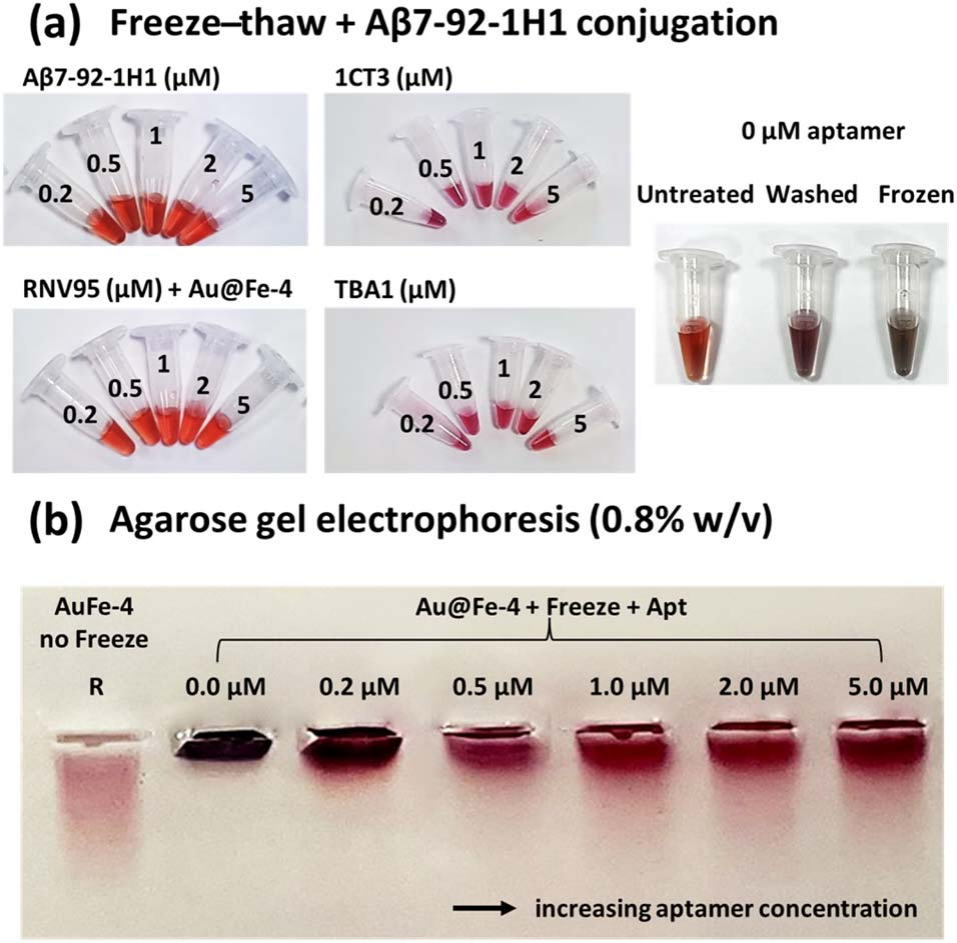
(a) Au@Fe-4 dispersions at a fixed nanoparticle concentration (0.32 mg mL^−1^), illustrating the effects of freeze-thaw cycles and aptamer conjugation (Aβ7-92-1H1 for Aβ42, RNV95 for Aβ40, 1CT3 for GFAP, and TBA1 for thrombin; concentrations: 0.0–5.0 µM). Reference Au@Fe-4 samples (0 µM aptamer) are included for comparison, shown untreated, after washing (following the same protocol as that for conjugated samples), and/or after freeze-thaw treatment. (b) Agarose gel electrophoresis (0.8% w/v) of Au@Fe-4 samples after freeze-thaw treatment in the absence (no freeze) or presence (+freeze) of the Aβ7-92-1H1 aptamer (0–5 µM).

To validate both the aptamer conjugation efficiency onto the Au@Fe-4 MNPs and the effect of conjugation on their stability, we analyzed Au@Fe-4 MNPs after conjugation with several concentrations of DNA aptamer Aβ7-92-1H1 by agarose gel electrophoresis (Fig. 6b). Au@Fe-4 MNPs without freezing-thawing treatment (sample R1) appear smeared, possibly due to innate size/stability polydispersity. After the freeze-thaw procedure nanoparticles without aptamer conjugates (0.0 µM aptamer concentration) are aggregated, an event which is determined by the significant motility impairment and color change (dark), when compared with the untreated MNPs. Freeze-thawed Au@Fe-4 MNPs incubated with increasing concentrations of the Aβ7-92-1H1 aptamer are also shown in Fig. 6 for aptamer's concentrations of 0.2, 0.5, 1, 2.0 and 5.0 µM. As the aptamer dose increased from 0.2 to 2 µM, a well-dispersed form of aptamer-functionalized magnetic nanoparticles (apt-MNPs) was observed, as the samples ran through the gel. Interestingly, the aptamer-conjugated Au@Fe-4 MNPs' preparations appeared significantly more dispersed than the untreated nanoparticles, verifying that aptamer modification stabilizes the Au@Fe-4 MNPs through the employed treatments (freeze-thaw, washes, and re-dispersions).

Likewise, the color of the nanoparticle dispersions in samples Au@Fe-4 MNPs + Freeze + Apt 0.0, 0.5, 1.0, and 2.0 µM, respectively, of Aβ7-92-1H1 (aptamer against Aβ_42_), turned more vibrant as the aptamer concentration increased (Fig. 6), further confirming a dose-dependent conjugation. Notably, the washing steps are capable of causing a slight aggregation of MNPs, as determined by the darker color of the sample with the 0.0 µM aptamer concentration, when compared with the untreated Au@Fe-4 MNPs (the reference sample R). However, when MNPs are functionalized with aptamers, washing procedures do not significantly affect their dispersion, as they retain a vibrant red color. The same analyses were also performed for the 3 other aptamers studied, and the representations in Fig. 6 were proven to be indicative of the general nature of aptamer-Au@Fe-4 nanoconjugates, producing similar dispersions and electrophoretic patterns. While visual inspection (Fig. 6a) provides a preliminary indication of stability, the precise quantification of aptamer loading for all four targets is detailed in Table S2 and qualitatively supported by DLS/UV-Vis data in Fig. S4 (SI).

To further validate the successful surface functionalization and investigate the impact of aptamer attachment on the nanoparticles' physical properties, DLS and UV-Vis spectroscopy were employed for all four studied aptamers (Fig. S4, SI). DLS measurements revealed a consistent increase in the hydrodynamic diameter of Au@Fe-4 MNPs following conjugation, confirming the formation of a stabilizing molecular coating. This was corroborated by UV-Vis spectra, which showed a characteristic redshift (3–5 nm) of the Surface Plasmon Resonance (SPR) peak. These findings, together with the gel electrophoresis data, provide robust qualitative evidence of effective conjugation across all aptamer sequences. This confirmed stability highlights the advantage of the Au@Fe-4 platform, which successfully synergizes high magnetic responsiveness with efficient plasmonic-based surface functionalization.

### 3.4 Optimized protocol of aptamer conjugation

**Fig. 7:**
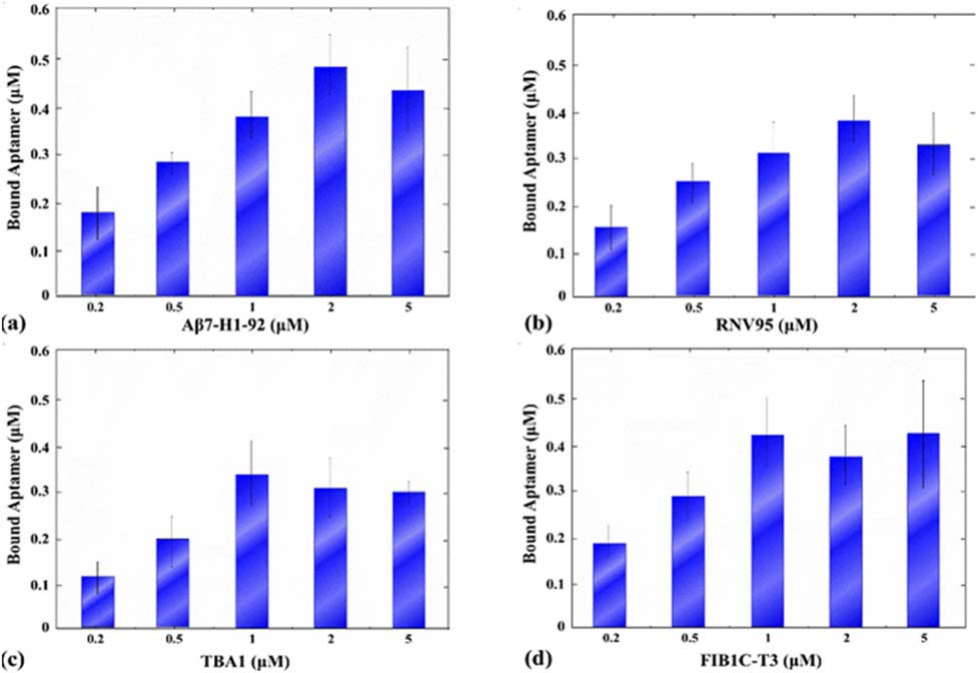
Quantitative analysis of DNA aptamer conjugation onto MNPs at a fixed nanoparticle concentration (0.32 mg mL^−1^) for four targets: (a) Aβ7-92-1H1, (b) RNV95, (c) TBA1, and (d) FIB1C-T3. The *y*-axis represents the absolute amount of the bound aptamer (µM) determined *via* fluorescence measurements of DTT-released FAM-labelled aptamers (*E*_x_: 490 nm; *E*_m_: 510–570 nm). For the exact numerical values and corresponding binding efficiency (%) at each concentration, please refer to Table S2 in the SI.

Quantitative analysis of aptamer loading onto Au@Fe-4 MNPs at a fixed nanoparticle concentration (0.32 mg mL^−1^) revealed a clear, concentration-dependent increase in bound oligonucleotide for all four employed aptamers—Aβ_42_ (Aβ7-92-1H1), Aβ_40_ (RNV95), thrombin (TBA1), and GFAP (FIB1C-T3). In each case, fluorescence measurements following DTT-mediated release of the aptamer demonstrated a steep rise in binding between 0.2 µM and 1 µM, with loading curves beginning to plateau between 2 µM and 5 µM (Fig. 7a–d). The detailed numerical parameters, including the calculated binding efficiency (%) for each input concentration, are provided in Table S2 of the SI. Regarding the aptamers Aβ7-92-1H1 and RNV95 that target the crucial AD-related peptides Aβ_42_ and Aβ_40_, saturation was reached at the initial employed concentration of 2 µM and was at approximately 0.49 µM and 0.38 µM, respectively. For the thrombin aptamer TBA1 and the GFAP aptamer FIB1C-T3, the saturation points were at 1 µM of initial aptamer, with final conjugation concentrations of 0.33 µM and 0.42 µM, respectively.

These quantitative trends were corroborated visually by the retention of the vibrant red color of nanoparticles' dispersions (Fig. 6), indicating that aptamer conjugation on Au@Fe-4 MNPs does not impair their stability and dispersion. Taken together, these data indicate that an aptamer concentration of 1–2 µM provides an optimal balance between efficient nanoparticle conjugation and the economical use of the oligonucleotide across diverse targets.

To provide structural insights into the molecular recognition mechanisms and corroborate the experimental affinity, we performed molecular docking simulations. The tertiary structures of the RNV95 and Aβ7-92-1H1 aptamers and the (TBA1) control were predicted using the 3dDNA Web Server.^66^ Putative active binding sites on the target proteins were identified using the SPPIDER server,^56^ followed by docking analysis *via* HADDOCK.

To systematically prioritize the most promising candidates, a high-throughput computational screening was performed across multiple AD-related targets. As summarized in Table S3 (SI), the binding score analysis revealed a clear hierarchy in binding potential. The beta-amyloid peptide (1 YT) exhibited the highest overall binding efficiency with a mean score of 5149.98 and a top 5% threshold of 6061.00, significantly outperforming other biomarkers such as pTau-217 (902.97) and GFAP (567.97). This quantitative ranking provided the structural rationale for focusing our experimental efforts on Aβ-targeted nanoconjugates, while also predicting the lower binding affinities observed for the other targets in subsequent assays.

The validity of the docking protocol was established using the TBA-thrombin complex as a benchmark, which yielded a high-affinity cluster (cluster 1) with a HADDOCK score of −67.1 ± 8.9 (*Z*-score −1.4). Notably, the aptamers proposed in this work exhibited binding profiles comparable to those of this gold standard. The interaction between Aβ7-92-1H1 and RNV95 displayed exceptional stability with a HADDOCK score of −56.1 ± 12.0 (*Z*-score −1.0). Furthermore, while the TBA benchmark presented a buried surface area (BSA) of 1400.4 ± 100.9 Å^2^, the amyloid-targeting aptamers demonstrated significantly more extensive interfacial contacts. The RNV95 complex revealed a massive BSA of 2278.4 ± 153.5 Å^2^, and the RNV95-Aβ_40_ complex (HADDOCK score −43.4 ± 38.5) showed a BSA of 1977.7 ± 286.2 Å^2^. Based on the analysis of the top-ranking clusters, these results suggest that while the TBA1 interaction is primarily electrostatically driven, the amyloid-targeting aptamers achieve high affinity through a deeper molecular encapsulation of the target peptide, as evidenced by their significantly larger buried surface areas (BSAs). This is further supported by the high-throughput binding score analysis (Table S3, SI), where Aβ-42 demonstrated a significantly higher mean binding score (5149.98) compared to the other targets like GFAP (567.97) and pTau-217 (902.97). This structural feature is a favorable trait for the specific detection of aggregation-prone biomarkers in complex matrices, justifying the selection of Aβ-42 as the primary target for our sensing platform.

## 4 Discussion

The Au@Fe nanoparticles synthesized in this study exhibited a controlled hydrodynamic size distribution (45–60 nm), uniform gold shell coverage, and tunable surface charge, which collectively ensured high colloidal stability and reproducible magnetic response. These structural and magnetic characteristics are critical for subsequent aptamer conjugation, as they dictate surface accessibility, interparticle interactions, and the efficiency of thiol–gold bonding. Optical characterization confirmed strong plasmonic activity, supporting the potential for optical readouts in biosensing applications. Overall, the synthesis strategy allowed precise tailoring of nanoparticle properties to balance magnetic manipulability, colloidal stability, and surface functionality for versatile biofunctionalization.

The hydrodynamic diameter of the optimized nanoparticles (∼45 nm) falls within the lower limit of reported Au@Fe nanostructures, while retaining excellent colloidal stability and structural uniformity. Literature values vary widely depending on the synthesis route and stabilization strategy.^67^ For instance, PEGylated Au@Fe nanoparticles prepared *via* co-precipitation typically reach hydrodynamic diameters of around 29 ± 4 nm after gold coating, with TEM core sizes near 24 nm and gold shell thicknesses of 11–15 nm.^68^ Janus-type Au@Fe nanoparticles synthesized through thermal decomposition often exhibit core sizes of 16–20 nm, while microemulsion-based Au@Fe systems yield ∼10 nm structures with thin (2 nm) shells.^69^ Methods such as the Brust–Schiffrin protocol can produce a broader size distribution, ranging from 5 up to 100 nm, depending on nucleation and growth conditions.^70^ Within this context, the ≈45 nm hydrodynamic size of Au@Fe-4 represents an advantageous balance between compact dimensions and robust colloidal stability, particularly when compared with larger citrate-stabilized Au-superparamagnetic iron oxide nanoparticle (Au-SPION) hybrids, which often exceed 70–100 nm due to secondary aggregation during the gold reduction step.^53–55^ The ζ-potential of the optimized Au@Fe nanoparticles (−73 mV) is more negative than that typically reported for Au@Fe systems, indicating exceptional colloidal stability and strong electrostatic repulsion between particles. In the work of Stein *et al.*,^55^ citrate-stabilized Au-SPION hybrids synthesized at pH 7.0 exhibited ζ-potentials of −48.0 ± 6.3 mV for citrate iron oxide nanoparticles, −43.5 mV for Au@Fe, and −48.6 mV for citrate-restabilized citrate Au@Fe nanoparticles—values that already reflect the beneficial stabilizing effect of citrate on iron oxide surfaces. In contrast, studies lacking citrate stabilization, such as the work of Carlos Caro *et al.*,^71^ often report significantly highly negative ζ-potential, ranging from approximately −4.89 mV to −10 mV, illustrating the poor electrostatic stabilization of non-citrate-coated Au@Fe nanoparticles and their tendency to aggregate.

The optimized magnetic behavior further aligns with, yet also distinctively improves upon, trends reported for gold-coated magnetic nanoparticles in the literature.^55^ The measured saturation magnetization (*M*_s_ ≈ 77 A m^2^ kg^−1^) is relatively high compared with previously published values for Au@Fe core–shell materials, where *M*_s_ often decreases significantly upon gold deposition. For instance, Elbialy *et al.*^54^ reported an *M*_s_ reduction from 58 to 19 A m^2^ kg^−1^ following gold coating, corresponding to only 32.8% retention of the original magnetic response, while citrate-stabilized Au-SPIONs reported by Mühlberger *et al.*^53^ exhibited even lower magnetization due to extensive citrate consumption and insufficient surface stabilization. In our case, a substantial fraction of the intrinsic magnetization of the Fe_3_O_4_ core is retained despite the presence of an optically active gold shell, suggesting that the gold growth mechanism in our synthesis forms a thin, continuous coating without excessively shielding the magnetic core. The magnetic behavior of the synthesized Au@Fe nanoparticles is consistent with the expected evolution of Fe_3_O_4_ when coupled with a gold phase. In Reguera's work^69^ on nano-dumbbells and gold-grown magnetic-plasmonic heterostructures, the iron oxide cores (∼16–20 nm) remained fully superparamagnetic, exhibiting no remanence or coercivity, with *M*(*H*) curves fitting well to a Langevin model and saturation magnetization values ranging from 45–70 emu g_Fe_^−1^. This regime is typical for highly diluted, single-domain iron oxide nanoparticles where interparticle interactions are minimized.

In contrast, the Au@Fe system developed here exhibits a small but measurable coercive field, which is expected for particles residing near the single- to multi-magnetic domain boundary, where dipolar interactions and local anisotropy contributions become significant. The presence of the gold phase, as also reported in other Au@Fe core–shell or decorated assemblies, contributes to a reduction in saturation magnetization due to the increased non-magnetic fraction and partial disturbance of magnetic coherence at the interface.

Taken together, the magnetic response of the synthesized Au@Fe nanoparticles aligns well with the established behavior of gold-modified iron oxide nanostructures: a preserved near-superparamagnetic character with suppressed magnetization and a small coercive field arising from size- and interaction-driven deviations from ideal single-domain behavior. This confirms the successful formation of hybrid architecture that retains magnetic responsiveness while enabling the plasmonic and functional capabilities imparted by the gold component.

In this study, we established and optimized a robust freezing-directed protocol for conjugating thiolated DNA aptamers onto core–shell Au@Fe magnetic nanoparticles and demonstrated its applicability across four AD-related biomolecular targets: amyloid-β_42_ (Aβ_42_), amyloid-β_40_ (Aβ_40_), thrombin (Thr), and glial fibrillary acidic protein (GFAP).^72–74^

Our findings provide insight into the key physicochemical parameters governing aptamer-nanoparticle interactions and underscore the versatility of the proposed conjugation strategy.

Building upon established thiol-gold conjugation protocols,^75^ we systematically assessed the influence of pH, ionic strength, washing conditions, and reagent concentrations on conjugation efficiency and robustness (Fig. 5).

The apparently contradictory effects of citrate when used during conjugation (Fig. 5a) *versus* when used as a wash (Fig. 5d) can be rationalized by well-known ligand-exchange and buffer-adsorption phenomena at Au surfaces. Acidic citrate (pH ≈ 3) reduces the effective negative surface charge and thus it weakens citrate adsorption, thereby facilitating the approach of the negatively charged aptamer and promoting initial thiol-gold ligand exchange and high apparent binding during the overnight conjugation step.^63,76,77^ However, as citrate–Au interaction is a well-known phenomenon and is also included in the synthetic pathway of Au@Fe-4 formulation, we cannot exclude the possibility that this effect could be more complex, based on other physicochemical phenomena, rather than a mere pH effect. Citrate ions and other buffer components can also be adsorbed competitively or re-equilibrate at the Au interface and therefore weakly displace the physiosorbed (non-chemisorbed) oligonucleotides during subsequent washing; mixed-monolayer studies and salt-aging experiments show that only chemisorbed thiol layers and properly packed monolayers remain after stringent washing, whereas loosely adsorbed DNA is removed.^65,78^ Taken together, these mechanistic insights explain why citrate promotes initial aptamer association but may reduce the retained aptamer when applied during wash steps, and they highlight the importance of optimizing both conjugation and post-conjugation washing conditions for stable Au–thiol bioconjugates. In contrast, washing steps performed in TE buffer (pH 8) preserved maximal aptamer retention while retaining nanoparticle colloidal stability, indicating that mildly basic conditions are advantageous during post-conjugation processing.

This pronounced pH dependence is consistent with prior reports highlighting the sensitivity of thiol–gold chemistry to the protonation state and surface charge effects, thereby reinforcing the importance of pH control in thiol-mediated functionalization strategies.^79^ Citrate molecules are expected to be present both at the magnetite–gold interface and on the external gold surface, where they act as stabilizing agents. However, due to the strong Au–S affinity, thiol-containing molecules can readily displace weakly bound citrate species and form stable Au–S bonds on the nanoparticle surface.

Nanoparticle concentration and ionic strength were found to modulate aptamer loading in a systematic and predictable manner. Increasing the MNP concentration up to 0.32 mg mL^−1^ resulted in enhanced conjugation efficiency, beyond which surface saturation limited further gains. Variations in ionic strength revealed a threshold-dependent response: moderate NaCl concentrations (30–100 mM) exerted minimal influence on aptamer binding, whereas higher salt levels (≥150 mM) led to a pronounced decrease in conjugation efficiency. This reduction is most plausibly attributed to salt-induced colloidal destabilization and electrostatic screening, which hinder aptamer access to the gold surface and promote nanoparticle aggregation—an effect well documented for gold- and iron oxide-based nanostructures at elevated ionic strengths.^80,81^

Across all four aptamers examined, a characteristic dose-dependent binding behavior was observed, marked by a steep increase in loading between 0.2 µM and 1 µM aptamer concentrations, followed by saturation beyond 2 µM (Fig. 7).

Notably, the thrombin aptamer TBA1 and the GFAP aptamer FIB1C-T3 approached maximal surface coverage at lower input concentrations (∼1 µM), whereas the Alzheimer's-related aptamers required higher concentrations (∼2 µM) to reach a comparable plateau. These distinct loading profiles likely arise from intrinsic sequence-dependent factors, including aptamer length, secondary structure stability, and steric accessibility at the nanoparticle surface. In particular, the Aβ7-92-1H1 and RNV95 aptamers are substantially larger than TBA1 and FIB1C-T3, necessitating higher initial concentrations to achieve equivalent surface coverage. Importantly, the convergence of all binding curves within the 1–2 µM range identifies this window as an effective and economical loading regime for subsequent assays. This observation is consistent with previous reports describing near-saturation behavior at comparable or lower concentrations for thiolated aptamers of similar length, such as LDL-specific sequences (∼40 residues).^82^

Additionally, our results demonstrate that conjugation of the Aβ7-92-1H1 DNA aptamer markedly enhances the colloidal stability of MNPs under freeze-thaw cycling and repeated washing stress. In the absence of aptamer functionalization, MNPs rapidly aggregated during freeze-thaw treatment, as evidenced by smeared electrophoretic bands and a pronounced darkening of the nanoparticle dispersion. By contrast, increasing aptamer concentrations from 0.2 to 5 µM progressively restored electrophoretic mobility and the characteristic red dispersion of the particles, indicating dose-dependent surface coverage and stabilization (Fig. 6a and b). This enhanced stability is most plausibly attributed to the combined electrostatic repulsion and steric hindrance conferred by the surface-bound DNA corona, which mitigates interparticle contact during ice crystal formation—an effect consistent with previous reports on freeze-driven DNA adsorption and nanoparticle stabilization mechanisms.^65,76,83^

Beyond freeze-thaw treatments, repeated washing steps alone were sufficient to induce partial aggregation of unmodified MNPs, whereas aptamer-functionalized particles consistently retained their characteristic red dispersion. This behavior highlights both the robustness of the Au–S anchoring chemistry and the protective role of the surface-bound DNA layer in preserving colloidal stability. Consistent with this interpretation, Li *et al.* systematically demonstrated that increased DNA packing density and multidentate thiol anchoring significantly enhance the thermal and chemical stability of DNA-AuNP conjugates under competitive and stress-inducing conditions.^42^ Extending these findings, our results show that even moderate aptamer surface densities (≥1 µM input concentration) are sufficient to retain nanoparticle stability against both thermal perturbations and repeated washing cycles. To further validate these observations, we are currently performing more sensitive photometrical and fluorometric analyses that specifically quantify both the ability and kinetics of aptamer-Au@Fe-4 conjugation. This is a crucial step towards our next goal: verifying the feasibility of these nanoconjugates in a biosensing platform for the diagnosis of AD and related neurodegenerative diseases.

Building on these findings, the versatility of the proposed platform is demonstrated by its capacity to support four distinct aptamer-MNP conjugates, each prepared independently to preserve sequence-specific folding, binding affinity, and optimal surface density. Notably, the RNV95 aptamer targeting Aβ_40_ was previously employed in colorimetric AuNP-based assays for the selective detection of low-molecular-weight Aβ_40_ oligomers, highlighting the diagnostic relevance of this recognition element.^50^ To the best of our knowledge, this work constitutes the first report of a unified gold-shell/iron-oxide-core nanoplatform capable of accommodating multiple target-specific DNA aptamers within a modular, single-sequence conjugation framework. By integrating the optical signature and colloidal robustness of gold shells with the magnetic manipulability of iron oxide cores, the resulting dual-function construct provides a promising foundation for magneto-optical, multiplex-ready nanobiosensors and theranostic systems targeting Aβ species, thrombin, and GFAP.

In conclusion, the optimized freezing-directed conjugation strategy enables reproducible and high-density aptamer functionalization of Au@Fe magnetic nanoparticles under mild and scalable conditions. Fine control over pH, ionic strength, and nanoparticle concentration allows adaptable loading across diverse aptamer sequences, paving the way toward next-generation diagnostic platforms and targeted nanotherapeutic applications.

## 5 Conclusions

A two-step aqueous synthesis of Au@Fe core–shell nanoparticles was established, enabling precise control over particle size, gold shell thickness, and surface charge. Structural, optical, and magnetic characterization confirmed the formation of homogeneous, magnetically responsive, and plasmonically active nanostructures, concluding in a model system for subsequent biofunctionalization.

Efficient conjugation of thiolated DNA aptamers targeting Alzheimer's disease-related biomarkers (Aβ_42_, Aβ_40_, thrombin, and GFAP) was achieved, as evidenced by concentration-dependent fluorescence binding profiles, while retaining nanoparticle stability and dispersibility. Fluorescence quantification revealed a concentration-dependent binding profile, confirming efficient and reproducible aptamer loading without compromising nanoparticle stability or dispersibility.

These findings demonstrate that Au@Fe hybrids provide a versatile dual-functional platform combining magnetic manipulability with optical signal transduction. The developed MNP-aptamer constructs hold strong promise for next-generation biosensing applications, particularly for the early, selective, and multiplexed detection of AD-associated biomarkers. Future work will focus on integrating these nanoconjugates into microfluidic or point-of-care analytical devices to translate their laboratory performance into practical diagnostic tools.

## Author contributions

Conceptualization: A. M., K. K., G. K., and E. E. T. Data curation: A. M., K. K., G. K., and E. E. T. Formal analysis: A. M., K. K., G. K., and E. E. T. Funding acquisition: M. A. Investigation: A. M., K. K., G. K., and E. E. T. Methodology: A. M., K. K., G. K., and E. E. T. Project administration: M. A. and A. A. P. Resources: M. A. and A. A. P. Software: C. B. and R. S.-C. Supervision: M. A. and A. A. P. Validation: A. M., K. K., G. K., and E. E. T. Visualization: A. M. Writing – original draft: A. M. Writing – review & editing: A. M., K. K., M. A., and A. A. P.

## Conflicts of interest

There are no conflicts to declare.

## Supplementary Material

NA-008-D6NA00021E-s001

## Data Availability

The data supporting this study are available in the Zenodo repository, including raw experimental data for ICP-OES concentration analysis, DLS hydrodynamic size distributions, UV-Vis absorption spectra, VSM magnetic measurements, and XRD diffraction patterns, at DOI: https://doi.org/10.5281/zenodo.17977203 Supplementary information (SI): additional supporting data, including TEM micrographs, magnetic hysteresis loops, DLS and XRD characterization, quantitative aptamer conjugation efficiency tables, and nanoparticle molecular weight calculations; Fig. S1–S4 and Tables S1–S3 mentioned in the text. See DOI: https://doi.org/10.1039/d6na00021e.
