# Supplementary material for: Biomarker-targeted functionalized magnetic nanoparticles: synthesis and aptamer conjugation optimization toward Alzheimer's disease biosensing
Source: Nanoscale Adv. 2026 Apr 6;8(10):3151–65. doi: 10.1039/d6na00021e (PMC13096835; doi:10.1039/d6na00021e)
Supplement: NA-008-D6NA00021E-s001 [file NA-008-D6NA00021E-s001.pdf]

Supplementary Information (SI) for Nanoscale Advances.
This journal is © The Royal Society of Chemistry 2026

Biomarker-Targeted Functionalized Magnetic Nanoparticles: Synthesis and Aptamer Conjugation Optimization Toward Alzheimer's Disease Biosensing

Antonios Makridis,*[a,b] Konstantina Kazeli,[a,b] Georgios Katsipis,[c,d] Eleni E. Tzekaki,[c,d] Anastasia A. Pantazaki[c,d], Cristian Bosch[e], Ricardo Simon-Carbajo[e] and Makis Angelakeris [a,b]

[a] *Magnetic Nanostructure Characterization: Technology & Applications, Centre for Interdisciplinary Research and Innovation, Aristotle University, 57001, Thessaloniki, Greece.*
[b] *Department of Condensed Matter and Materials Physics, School of Physics, Aristotle University of Thessaloniki, 54124, Thessaloniki, Greece.*
[c] *Laboratory of Neurodegenerative Diseases, Centre for Interdisciplinary Research and Innovation, Aristotle University, 57001, Thessaloniki, Greece.*
[d] *Laboratory of Biochemistry, Department of Chemistry, Aristotle University of Thessaloniki, Thessaloniki, 54124, Greece.*
[e] *Ireland's Centre for Artificial Intelligence (CeADAR), University College Dublin, Belfield, D04 V2N9 Dublin, Ireland.*

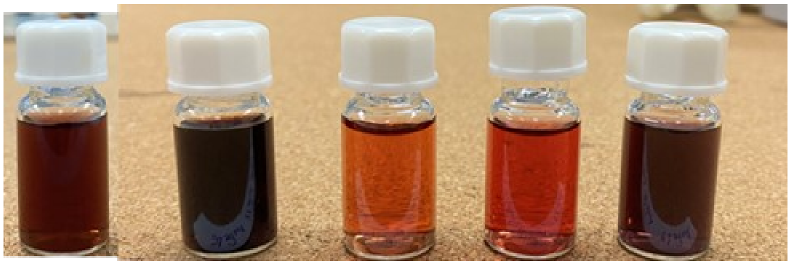

Figure S1. *Digital photograph of the synthesized Au@Fe nanoparticle dispersions (Au@Fe-1 to Au@Fe-5, from left to right). The color gradient from dark brown to vivid wine-red illustrates the influence of the gold precursor concentration and post-synthesis treatments on the optical properties and shell formation of the nanoplatforms.*

The structural architecture and morphology of the optimized Au@Fe-4 nanoplatform were investigated using Transmission Electron Microscopy (TEM). As shown in Figure S1 (Supporting Information), the micrographs provide definitive structural proof of a decorated hybrid morphology. The high-contrast regions (darker spheres) correspond to the gold nanoparticles, which are effectively integrated with the magnetite clusters (lighter grey regions). This hybrid configuration confirms that the two phases are physically and chemically coupled into a single nanostructure, rather than existing as a simple physical mixture of independent particles. The primary particle size observed across multiple TEM fields ranges approximately between 35 and 50 nm, demonstrating the successful formation of stable, non-aggregated hybrid assemblies.

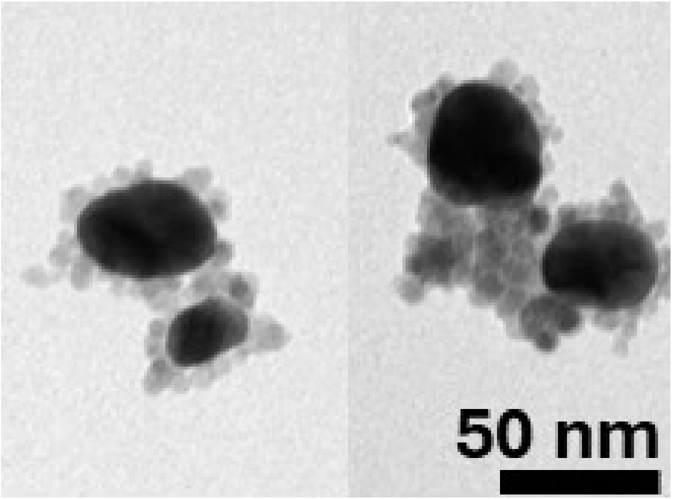

Figure S2. *TEM images of the optimized Au@Fe-4 MNPs. The image confirms a hybrid Au–Fe_3O_4 architecture where the two phases are closely integrated, forming a magnetically responsive and plasmonically active nanostructure.*

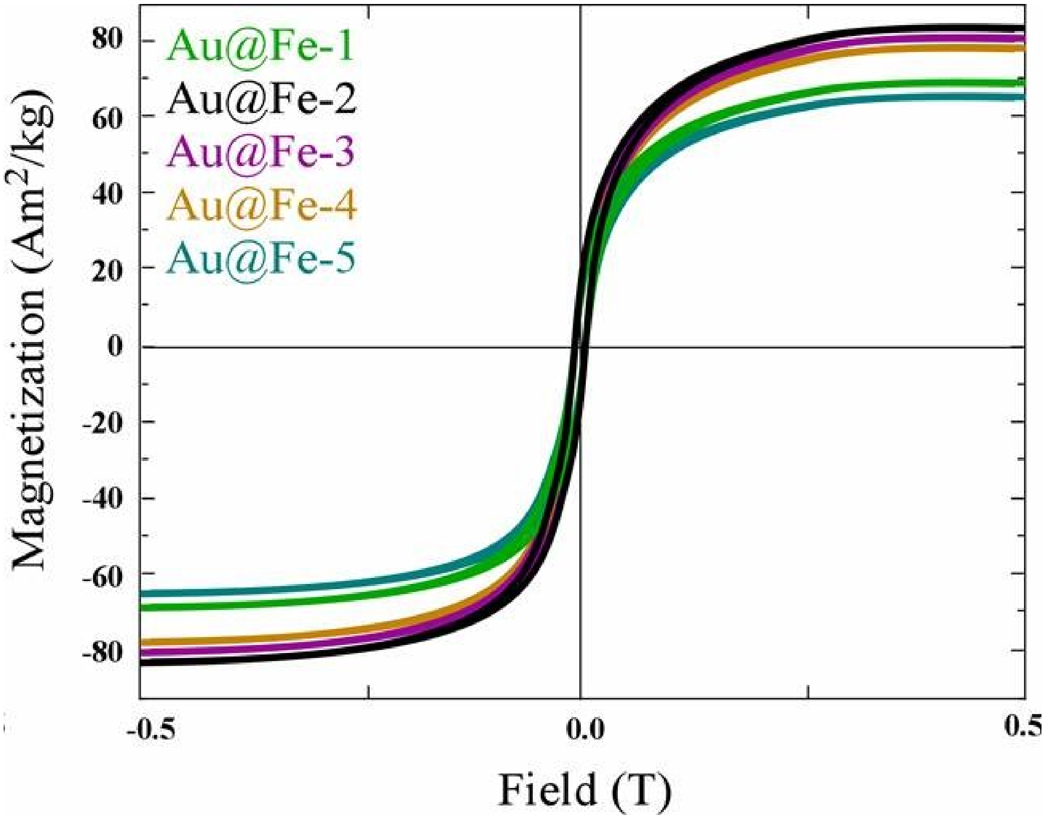

Figure S3.

Magnetic hysteresis loops of the five synthesized Au@Fe nanoparticle formulations measured at room temperature. All samples exhibit very similar magnetic behavior, confirming that the

magnetite cores were produced using the same synthesis protocol. Small variations in the saturation magnetization are attributed to differences in the amount of $HAuCl_4$ used during the gold deposition step, which resulted in slightly different Au/Fe ratios among the samples. Since the saturation magnetization is expressed per gram of total nanoparticle mass (emu g^{-1}), the presence of different fractions of non-magnetic gold leads to minor variations in the measured magnetic response.

Table S1. *Comparative physicochemical characterization of the synthesized Au@Fe nanoparticle series (Au@Fe-1 to Au@Fe-5). The table summarizes the hydrodynamic diameters (D_h) and ζ-potential values obtained by Dynamic Light Scattering (DLS), alongside the relative intensities of the Au (111) diffraction peaks from XRD analysis and the macroscopic visual appearance of the colloidal dispersions.*

Sample	***Hydrodynamic Diameter (nm)***	***ζ-Potential (mV)***	***Au (111) XRD Intensity***	***Visual Appearance***
Au@Fe-1	160	68	*Low*	*Brownish*
Au@Fe-2	73	-58	*Moderate*	*Reddish-brown*
Au@Fe-3	33	*-60*	*Low*	*Light Red*
Au@Fe-4	***45***	***-73***	***High***	***Wine-Red***

Au@Fe-5	70	*-68*	*Very High*	*Deep Purple*

Table S2. *Quantitative parameters and binding efficiency (%) of DNA aptamer conjugation onto Au@Fe-4 MNPs for all four studied targets.*

Target Aptamer	***Input Aptamer (µM)***	***Bound Aptamer (µM)***	***Binding Efficiency (%)***
Aβ7-92-1H1 ($A\beta_{42}$)	***0.2***	***0.16***	***80.0%***
	0.5	***0.32***	***64.0%***
	1.0	***0.42***	***42.0%***
	2.0	***0.49***	***24.5%***
	5.0	***0.51***	***10.2%***
RNV95 ($A\beta_{40}$)	***0.2***	***0.14***	***70.0%***
	0.5	***0.28***	***56.0%***
	1.0	***0.35***	***35.0%***
	2.0	***0.38***	***19.0%***
	5.0	***0.40***	***8.0%***
TBA1 (Thrombin)	***0.2***	***0.12***	***60.0%***
	0.5	***0.22***	***44.0%***
	1.0	***0.30***	***30.0%***
	2.0	***0.35***	***17.5%***

	5.0	***0.37***	***7.4%***
FIB1C-T3	***0.2***	***0.13***	***65.0%***
(GFAP)	***0.5***	***0.25***	***50.0%***
	1.0	***0.33***	***33.0%***
	2.0	***0.40***	***20.0%***
	5.0	***0.42***	***8.4%***

Table S3: *Statistical summary of binding scores for candidate aptamers – Summary of mean, median, standard deviation, minimum, maximum scores, and top 5% score thresholds for aptamers targeting TBA, Aβ-42 peptide, GFAP and pTau-217.*

Target Protein	Mean Score	Median Score	Std. Deviation	Min. Score	Max. Score	Top 5% Threshold
**Beta-amyloid (1YT)**	5149.98	5049.27	479.05	4524.15	7642.17	6061.00
**pTau-217 (6XLI)**	902.97	882.72	86.92	798.59	1410.44	1071.88
**TBA (1HUT)**	855.93	834.12	81.26	760.84	1295.33	1021.71
**GFAP (6A9P)**	567.97	556.80	40.60	520.13	795.25	647.64

S1. Nanoparticle Molecular Weight Calculation

To calculate the molar concentration of the Au@Fe nanoparticles, we estimated the mass of a single nanoparticle (m_{NP}) based on its volume and composition:

1. *Volume (V_{NP}): For a spherical particle with an average radius (r) of 22.5 nm (from DLS):*

$$V_{NP} = \frac{4}{3}\pi r^3 \approx 4.77x10^{-17} cm^3$$

2. *Density (ρ_{total}): Calculated as a weighted average based on mass fractions:*

   - Fe_3O_4*: 55%* ($\rho = 5.17\ g/cm^3$)
   - *Au: 35%* ($\rho = 19.3\ g/cm^3$)
   - *Citric acid: 10%* ($\rho \approx 1.6\ g/cm^3$)
     Estimated average density: $\rho_{total} \approx 7.9\ g/cm^3$

3. *Mass* (m_{NP})*:* $m_{NP} = V_{NP} x \rho_{total} \approx 3.77x10^{-16}g$

4. *Molecular Weight* (M_r)*: Using Avogadro's number* (N_A)*:* $M_r = m_{NP} x N_A \approx 2.27x10^{8}\ g/mol$

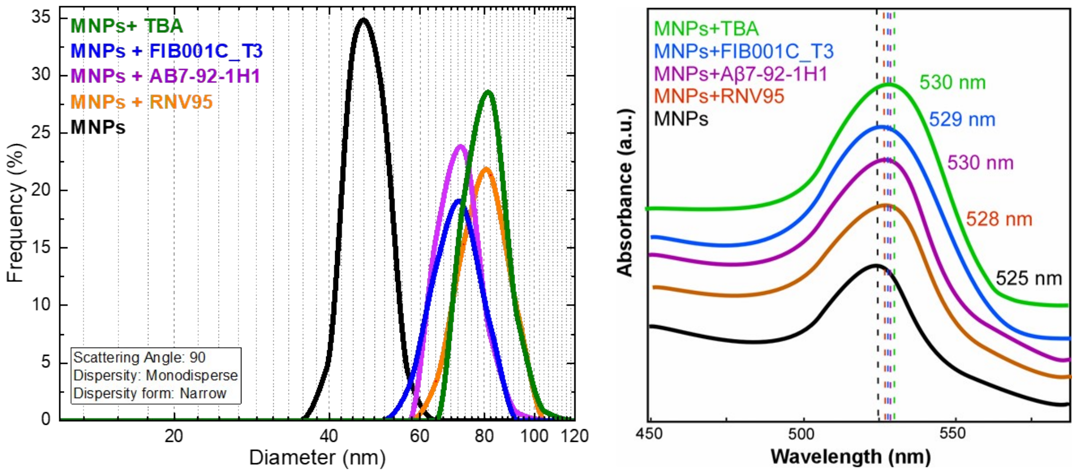

Figure S4. *Qualitative validation of conjugation for all four studied aptamers (RNV95, Aβ7-92-1H1, FIB1C-T3, and TBA1) onto Au@Fe-4 nanoparticles. (a) Dynamic Light Scattering (DLS) measurements showing the increase in the hydrodynamic diameter (size distribution) of the MNPs after functionalization with the selected aptamers (RNV95, Aβ7-92-1H1, FIB1C-T3, and TBA1). (b) UV-Vis absorption spectra demonstrating the characteristic redshift of the Surface Plasmon Resonance (SPR) peak from 525 nm (bare Au@Fe-4) to 528–530 nm upon successful aptamer attachment, indicating changes in the local refractive index at the nanoparticle interface.*
